# Mutation of *TP53*, translocation analysis and immunohistochemical expression of MYC, BCL-2 and BCL-6 in patients with DLBCL treated with R-CHOP

**DOI:** 10.1038/s41598-018-33230-3

**Published:** 2018-10-04

**Authors:** Pekka Peroja, Mette Pedersen, Tuomo Mantere, Peter Nørgaard, Jenni Peltonen, Kirsi-Maria Haapasaari, Jan Böhm, Esa Jantunen, Taina Turpeenniemi-Hujanen, Katrin Rapakko, Peeter Karihtala, Ylermi Soini, Kaija Vasala, Outi Kuittinen

**Affiliations:** 1 Department of Oncology and Radiotherapy, Cancer and Translational Medicine Research Unit, University of Oulu and Medical Research Center, Oulu University Hospital, Oulu, Finland; 2 Department of Pathology, Herlev and Gentofte University Hospital, University of Copenhagen, Herlev, Denmark; 3 Laboratory of Genetics, Northern Finland Laboratory Centre NordLab Oulu, Oulu, Finland; 4 Department of Pathology, University of Oulu and Medical Research Center, Oulu, Finland; 5 Department of Pathology, Central Finland Central Hospital, Jyväskylä, Finland; 6 Department of Medicine, University of Eastern Finland/Clinical Medicine, Kuopio University Hospital, Siun Sote -North Carelia Central, Kuopio, Finland; 7 Department of Pathology, Kuopio University Hospital, Kuopio, Finland; 8 Department of Oncology and Radiotherapy, Central Finland Central Hospital, Jyväskylä, Finland; 9 University of Eastern Finland, Faculty of Health Medicine, Institute of Clinical Medicine Oncology, Kuopio University Hospital, Kuopio, Finland

## Abstract

Diffuse large B-cell lymphoma (DLBCL) is an aggressive lymphoma with diverse outcomes. Concurrent translocation of *MYC* and *BCL-2* and/or *BCL-6*, and concurrent immunohistochemical (IHC) high expression of MYC and BCL-2, have been linked to unfavorable treatment responses. *TP53*-mutated DLBCL has also been linked to worse outcome. Our aim was to evaluate the aforementioned issues in a cohort of 155 patients uniformly treated with R-CHOP-like therapies. We performed direct sequencing of *TP53* exons 5, 6, 7 and 8 as well as fluorescence *in-situ* hybridization (FISH) of *MYC*, *BCL-2* and *BCL-6*, and IHC of MYC, BCL-2 and BCL-6. In multivariate analysis, *TP53* mutations in L3 and loop-sheet helix (LSH) associated with a risk ratio (RR) of disease-specific survival (DSS) of 8.779 (p = 0.022) and a RR of disease-free survival (DFS) of 10.498 (p = 0.011). In IHC analysis BCL-2 overexpression was associated with inferior DFS (p = 0.002) and DSS (p = 0.002). DLBCL with BCL-2 and MYC overexpression conferred inferior survival in all patients (DSS, p = 0.038 and DFS, p = 0.011) and in patients with non-GC phenotype (DSS (p = 0.013) and DFS (p = 0.010). Our results imply that in DLBCL, the location of *TP53* mutations and IHC analysis of BCL-2 and MYC might have a role in the assessment of prognosis.

## Introduction

Diffuse large B-cell lymphoma (DLBCL) is the most common type of lymphoma in the western countries and is clinically heterogeneous, with subsets of patients having diverse prognoses^1^. Gene expression profiling (GEP) has been used to classify DLBCL into subgroups by cell of origin (COO): germinal center (GC), activated B-cell (ABC) and a third type which cannot be classified into either categories^2^. Owing to the high costs of GEP and the requirement of fresh tissue samples, surrogate methods to classify DLBCL into COO subgroups have been developed, e.g. Hans’s IHC algorithm that classifies DLBCLs into two subgroups, GC and non-GC, which includes ABC and the third type^2^. Double-hit (DH) lymphomas, defined as lymphomas with *MYC* translocation combined with BCL-2 or BCL6 translocation, are among the most aggressive variants. In the newly revised WHO classification, DH lymphomas were classified in a new class of high-grade B-cell lymphoma^3^. Double-expressor (DE) DLBCLs (DLBCLs with high protein expression of MYC and BCL-2 but without translocation) were included in the not-otherwise-specified (NOS) category, but were implied to have negative prognostic significance^3^. A diversity in biology and clinical outcome exists within the individual categories. DH lymphomas are usually clinically very aggressive with poor responses to first-line treatments and with short remissions.

The *TP53* tumor suppressor gene located at chromosome region 17p13.1 encodes the p53 protein, which is involved in the regulation of cell cycle, DNA repair, apoptosis, and senescence after various stress signals, such as DNA damage and inflammation^4^. Loss of p53 function allows proliferation of cells with DNA damage and promotes neoplasia in transgenic p53-null mice^5^. Wild-type p53 functions as a cell-cycle checkpoint and a sensor of DNA damage in the cell^6^, and new functions keep emerging such as a role as a suppressor of inflammation^7^, and regulation of glucose metabolism^8^.

The *TP53* gene is mutated in about 20% of cases of DLBCL^9^, and most of the published mutations affect p53-DNA interactions, resulting in a partial or complete loss of transactivation functions^10^. *TP53* differs from other tumor suppressor genes in its mode of inactivation. While most tumor suppressor genes are inactivated by mutations leading to absence of protein synthesis or production of a truncated protein, more than 80% of *TP53* alterations are missense mutations that lead to the synthesis of a stable full-length protein^11^. The location of the resulting amino-acid substitution is usually within the central DNA-binding domain (DBD) of p53, resulting in a loss of DNA-binding activity with consequent failure to transcriptionally activate target genes. The most commonly mutated areas in the DBD are loop-sheet-binding helices (LSHs) L2 and L3. Various mutations have different consequences for the function of the p53 protein^12,13^. Some mutations are associated with a loss of function and others with a gain of function.

Only a small number of studies combining *TP53* mutation analysis, translocation data and double-expressor status in DLBCL have been published^14^. The results of previous studies imply that patients with combined mutation of *TP53* and double-hit translocation fare poorly^15^. Our series is one of the largest investigated to date. In the present study, we pursue the clinical importance of *TP53* mutation types combined with translocation and IHC data in patients with DLBCL.

## Results

### *TP53* mutation

Patient characteristics are summarized in Table 1 with comparison between wild-type (WT) and mutated *TP53*. Out of 155 patient samples all exon sequencings were successful in 80 samples (51.6%). In 26 (16.8%) samples all but one exon were successful. Sequencing was either unsuccessful in more than one exon or totally unsuccessful in 49 samples (31.6%). Nine missense mutations with eight non-functional and one partially functional mutation in *TP53* were detected in our patient material. One silent mutation with a synonymous protein product was also detected. The mutations are presented in Table 2. The total mutation frequency was detected 9.6%. In patient material with successful sequencing, 3-year DFS with mutated *TP53* was 66.7%, compared with WT *TP53*, 75.1% (p = 0.494). When comparing 3-year DSS values, the figure for those with mutated *TP53* was 66.7% versus WT *TP53*, 83.2% (p = 0.268). All three lymphoma-related deaths in patients with *TP53* mutations were due to primary refractory disease. No relapses were detected in patients with mutated *TP53* if initial treatment was successful (WT *TP53*, 132 patients with 26 relapses and in cases of mutated *TP53*, 6 patients with 0 relapses, p = 0.594). Structural analysis of mutations showed that two different mutations were present in LSH motifs, one in L3 and the other mutations were localized in β-sheets.

**Table 1:** Baseline and treatment characteristics.

		WT P53 (n/%)	Mutated P53 (n/%)	p-value	All patients (n/%)
Eastern Cooperative Oncology Group performance status	2, 3 or 4	4/6	2/22	0.136	20/13
Age	Over 60	45/63	8/89	0.260	98/63
Lactate dehydrogenase	High	39/55	6/67	0.724	88/57
Gender	Female	43/58	4/44	0.492	72/47
Stage	III–IV	36/51	5/56	1.000	81/53
Extranodal involvement	>1	7/10	4/44	0.018	27/17
B-symptoms	Yes	31/44	2/22	0.291	62/40
International prognosis index	0–1	30/43	1/11		59/38
	2–3	37/53	5/56		75/48
	4–5	3/4	3/33	0.008	18/11
Rituximab		71/100	9/100	1.000	155/100
Treatment response	Complete or partial	68/94	6/67		138/89
	Progressive disease	4/6	3/33	0.028	17/11
Mortality	Death from lymphoma	14/20	3/33		35/23
	Death from other cause	8/11	0/0	0.725	16/10
BCL-2 translocation		7/12	2/25	0.278	15/10
BCL-6 translocation		11/18	1/11	1.000	22/14
MYC translocation		1/2	1/11	0.342	11/7
Double hit		1/2	1/13	0.220	7/5
BCL-2 high expression		26/43	4/44	1.000	59/38
BCL-6 high expression		27/44	7/78	0.080	56/36
MYC high expression		29/48	5/56	0.734	63/41
Double expressor		18/30	1/13	0.720	34/22
Germinal center		24/34	3/33	0.302	56/36

**Table 2:** *TP53* mutations.

Case number	Exon	Mutation DNA	Mutated protein	Mutation type	TA class	Gain of function	Dominant negative activity	Structural motif
99	5	c.469G > A	V157I Val > Ile	missense	pF	NA	NA	β-sheets
92	5	c.486C > T	I162I Ile > Ile	silent	NA	NA	NA	β-sheets
47	7	c.707A > G	Y236C Tyr > Cys	missense	NF	NA	Yes35	β-sheets
100	7	c.726C > G	C242W Cys > Trp	missense	NF	NA	NA	L3
88	7	c.751A > C	I251L Ile > Leu	missense	NF	NA	NA	β-sheets
82	7	c.772G > A	E258K Glu > Lys	missense	NF	NA	Yes35	β-sheets
33	8	c.797G > A	G266E Gly > Glu	missense	NF	Yes (p73β interference)36	No36	β-sheets
105	8	c.809T > C	F270S Phe > Ser	missense	NF	Yes (p73β interference)36	No36	β-sheets
74	8	c.817C > G	R273G Arg > Gly	missense	NF	NA	Yes35	LSH
40	8	c.818G > A	R273H Arg > His	missense	NF	Yes (growth advantage, drug resistance)37	Yes37	LSH

**Figure 1:**
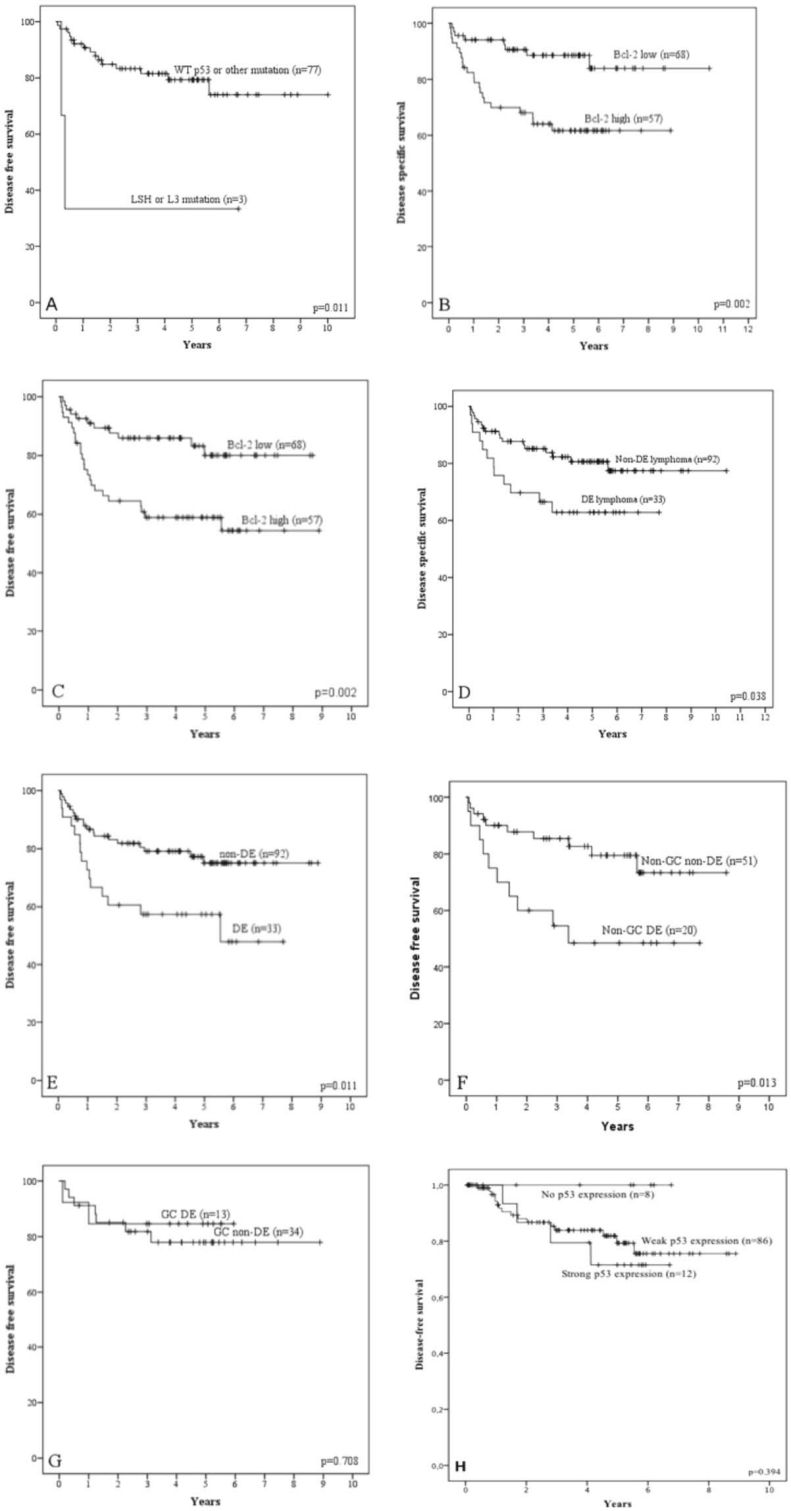
Survival figures. (**A**) LSH or L3 versus wild type p53 and other mutation DFS. (**B**) BCL-2 DSS. (**C**) BCL-2 DFS. (**D**) Double-expressor DSS. (**E**) Double-expressor DFS. (**F**) Double-expressor non-GC DFS. (**G**) Double-expressor GC DFS. (**H**) Immunohistochemical expression of p53.

Mutations in LSH and L3 motifs predicted 3-year DSS and DFS (3-year DSS 33.3% versus 83.3% in WT and β-sheet-mutated *TP53*, p = 0.011, and 3-year DFS 33.3% versus 75.8% in WT and β-sheet-mutated *TP53*, p = 0.027). Survival data is shown in Fig. 1. Despite very low number of patients and events, in multivariate analysis mutation of LSH and L3 remained an independent prognostic variable as the relative risk of death from lymphoma was 8.779 (95% CI, 1.377 to 55.972, p = 0.022). LSH and L3 mutations were also independent prognostic factors for DFS (RR 10.498; 95% CI, 1.710 to 64.449, p = 0.011). Results should be considered suggestive and with caution due to low numbers in the subgroups.

### Translocations

**Figure 2:**
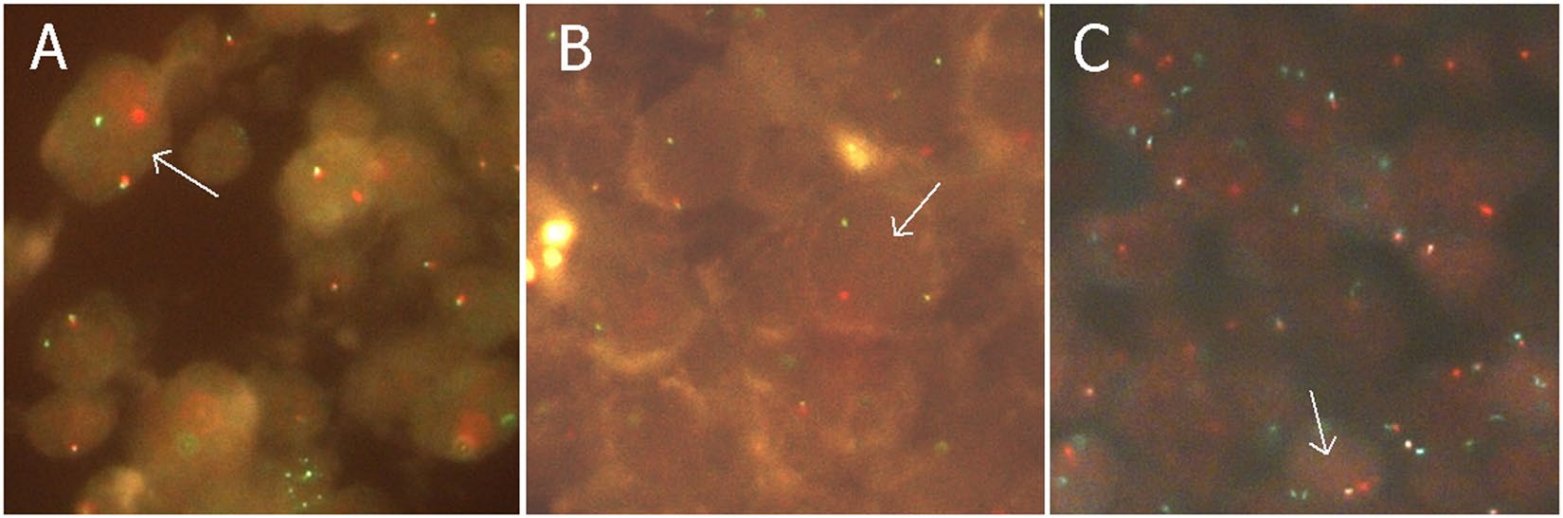
Gene translocations in DLBCL measured by fluorescent *in situ* hybridization (FISH). Composite photomicrograph with sections from representative 1 mm tissue microarray cores hybridized with dual color split FISH probes. A yellow fusion signal and red and green split signals in a cell are indicative of gene translocation (arrows). For quantitative analysis the focus must be continuously adjusted hence photographic reproduction is somewhat inaccurate. (**A**) *CMYC*. (**B**) *BCL6*. (**C**) *BCL2*.

FISH for *MYC, BCL-2* and *BCL-6* translocation was successful in 128/155 cases (82.6%) (Fig. 2). *MYC* translocations were detected in 11 (8.6%), *BCL-2* translocations in 15 (11.7%), *BCL-6* translocations in 22 (17.2%) and DH translocations in seven (5.5%) of the 128 cases. Three patients (2.3%) had DH with *BCL-2*, three patients (2.3%) DH with *BCL-6* and one (0.8%) patient had triple-hit lymphoma. MYC translocation did not correlate with any clinical factor. *BCL-2* translocation was associated with younger age (p = 0.05). None of the *BCL-2* translocation cases were of non-GC phenotype (p = 0.0000002). DH status showed a positive correlation with extranodal disease (p = 0.014).

*MYC* translocation predicted neither DFS nor DSS (3-year DFS 70.0% versus 73.5%, p = 0.981 and 3-year DSS 70.0% versus 81.1%, p = 0.687). The figures for *BCL-6* translocation were: 3-year DFS 79.1% versus 72.1% (normal BCL-6; p = 0.480) and 3-year DSS 79.1% versus 80.2% (p = 0.845). *BCL-2* translocation predicted neither DSS nor DFS (3-year DSS 73.3% versus 80.7%, p = 0.639 and 3-year DFS 60.0% versus 74.7%, p = 0.211). DH status had no prognostic value, as 3-year DFS was 83.3% versus 72.3% (p = 0.527) and 3-year DSS was 83.3% versus 79.6% (p = 0.695).

### Immunohistochemistry

**Figure 3:**
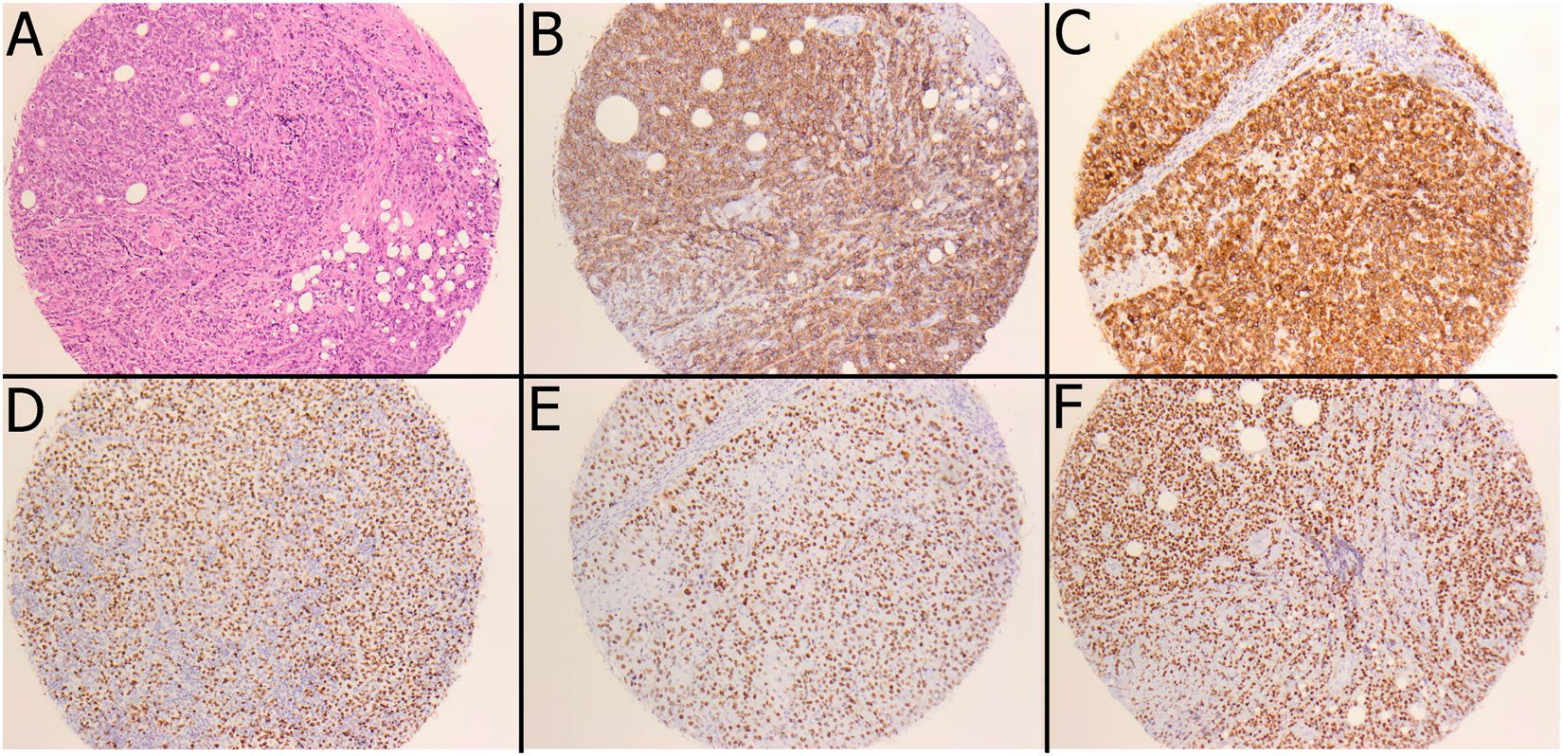
Protein overexpression in DLBCL measured by immunohistochemistry. Composite photomicrograph of representative 1 mm tissue microarray cores. The MYC-, BCL6- and p53 staining patterns are nuclear whereas CD20 shows membranous - and BCL2 cytoplasmic staining patterns. (**A**) Hematoxylin-eosine staining. (**B**–**F**) Immunohistochemical stainings (**B**) CD20. (**C**) BCL2. (**D**) BCL6. (**E**) MYC. (**F**) p53.

IHC GC and non-GC phenotyping was successful in 141 (91.0%) out of 155 samples. IHC evaluation of MYC and BCL-2 was possible in 128 (82.6%) samples and evaluation of BCL-6 in 129 (83.2%) (Fig. 3). A non-GC phenotype was associated with a trend towards worse DSS (3-year DSS 74.6% versus 83.5%, p = 0.123). High MYC expression correlated with an intermediate IPI score, compared with low- and high-risk scores (p = 0.007). High BCL-6 expression was associated with GC phenotype (p = 0.011).

High MYC (cut-off value 40%) or BCL-6 expression did not predict survival. High expression of BCL-2 was associated with worse DFS and DSS (3-year DFS 58.9% versus 85.9%, p = 0.002 and 3-year DSS 68.0% versus 90.6%, p = 0.002). Patients with DE lymphoma had worse 3-year DFS (57.4% versus 79.1%, p = 0.011) and worse 3-year DSS (66.5% versus 85.2%, p = 0.038). DE did not predict survival in patients with the GC phenotype, but DE status did predict both DSS and DFS in patients with the non-GC phenotype (3-year DSS 54.5% versus 85.4%, p = 0.013 and 3-year DFS 49.5% versus 76.2%, p = 0.010). When using cut-off value of 70% for MYC IHC same correlations were found albeit with better p values.

Immunohistochemical p53 expression was associated with *TP53* mutation (p = 0.00017) (Table 3). The sensitivity of high p53 expression to find *TP53* mutated cases was 55.6% and specifity 90.8%, respectively. Corresponding positive and negative predictive values were 31.3% and 96.4%, respectively. p53 immunohistochemical expression did not associate with traditional prognostic factors of DLBCL (performance status, IPI, stage, extranodal involvement) nor survival.

**Table 3:** Immunohistochemical p53 expression associates with *TP53* mutation (p = 0.00017).

	Immunohistochemical p53 expression		
	No expression	Low expression	High expression
*TP53* mutation detected	0 (0%)	4 (44%)	5 (56%)
*TP53* mutation not detected	3 (3.8%)	71 (89.0%)	6 (7.5%)

### Associations between the studied parameters

Only two patients had concurrent *TP53* mutation and *BCL-2* translocation. *TP53* mutations were located in LSH and L3 motifs (p = 0.021). These patients with concurrent *BCL-2* translocation and *TP53* mutation had very aggressive primary refractory DLBCL with a dismal outcome (mean DSS only 3 months, p = 0.000000002). There were no other associations between *TP53* mutation and translocations. High MYC expression was associated with *BCL-2* translocation (p = 0.002). *MYC* translocation was associated with high MYC expression (p = 0.030). *MYC* translocation was more common in *BCL-6* translocated lymphomas (p = 0.022). High BCL-2 expression was associated with *BCL-2* translocation (p = 0.011). BCL-2/MYC DE was significantly more common among patients with *BCL-2* translocation, as 10 out of a total of 15 *BCL-2* -translocated lymphomas had DE and 24 out of a total of 111 without *BCL-2* translocation were DE (p = 0.001).

## Discussion

*TP53* mutations and DH with *MYC* and *BCL-2* have been linked to inferior survival in patients with DLBCL^16–20^. Only a few studies have reported simultaneous analysis of genetic alterations and immunohistochemical protein expression of these genes^21–23^. Here we report a study describing p53 mutations, MYC, BCL-2 and BCL-6, translocations and immunohistochemical expression in a cohort of 155 newly diagnosed DLBCL cases. In the present study mutations of *TP53* in LSH and L3 motifs were the only mutation types that had a strong association with poor survival. Overall, *TP53* mutations were not associated with survival. High BCL-2 expression and DE status also had negative prognostic impact. DH translocation did not predict survival, nor did individual translocations. Patients were however few and results should be considered suggestive, interpreted with care and need further validation.

DH status has been established as a major survival predictor in DLBCL, and in the recently revised WHO classification DH DLBCL is categorized as an entity of its own among high-grade lymphomas (HGL)^18,22^. Many studies have shown that patients with *BCL-2* and *MYC* DH translocation show poor responses to treatment. However, some recent reports have not been able to confirm the very poor prognosis of this group. These discrepancies have been recently presented in a comprehensive review of the literature published by Rosenthal & Yunes^24^. Moreover, to describe further the biological impact of these translocations it been suggested that patients with *MYC* translocations should be substratified according to translocation partner^23,25–28^. The issue is further complicated by the fact that MYC/BCL-2 and MYC/BCL-6 double hit lymphomas seems to be biologically distinct and probably should be addressed separately^24^. One study has shown that IHC analysis of BCL-2 and MYC expression might have more prognostic impact than FISH alone^18^. This implies that mechanisms other than translocation affect protein expression, which is also supported by the fact more patients have high MYC protein expression than translocation. In the present material, DH status and translocations did not predict survival. This might be due to the rarity of these cases. In the present series limited number of patients with DH lymphoma did not allow for substratification according to *MYC* translocation partner gene. In contrast to this, IHC predicted worse survival in the BCL-2 expression group and in the DE group using cut-off values established in the previous studies of DE^29^.

In this study we used cut-off value of 40% for MYC positivity, which is used in most previous studies. Work by Ambrosio *et al*. including a large series of 753 patients with aggressive B-cell lymphoma suggested that cut-off value of 70% might be able to better define the true poor prognosis group of DE lymphomas^30^. We repeated our analyses with this higher cut-off value also. This change increased the statistical power of established correlations but still we did not find correlations with survival. This discrepancy with the results of Ambrosio *et al*. may be explained by our smaller cohort.

The mutation frequency of *TP53* is considered to be about 20% in *de novo* DLBCL^19,20^. Earlier studies of DLBCL have shown that most mutations occur in hot-spot regions, and mutations in LSH and L3 are associated with worse prognosis, while patients with L2 mutations show survival similar to those in WT groups. In a study by Young *et al*. in the pre-rituximab era concerning a cohort of 477 patients, 102 of the DLBCL cases were *TP53*-mutated. Mutations in LSH and L3 were associated with worse survival and *TP53* mutations in the DBD region were considered markers of poor prognosis^19^. In a later study by Xu-Monette *et al*. a rituximab-treated cohort of 506 patients was studied. Of these, 112 patients with mutation of *TP53* were detected and mutation was associated with worse prognosis. The study also established IHC-detected p53 as a suitable surrogate marker of mutation. A cut-off value of 50% quantified patients into a probable *TP53* mutation group and IHC of p53 was shown to have prognostic potential. Deletion of *TP53* was not associated with poor prognosis, only point mutations^20^. These studies, as well as other studies performed at rituximab era, established that mutations at the DBD region of *TP53* were prognostic in regards to survival in DLBCL, regardless of treatment^14,21,22^.

In the present study, LSH and L3 mutations of *TP53* were associated with poor survival. The other *TP53* mutations were located in β-sheets (in non-DNA-binding domains) and did not predict survival. LSH and L3 mutations of *TP53* mutations were however only detected in three patients and the statistical analysis should be considered with care. Moreover, while the value of p53 pathway in carcinogenesis is evident, the big picture seems to be much more complicated and cover a broader issue than just p53 gene mutations. p53 pathway is a complexed one with over 50 genes and proteins affected. Several genetic events commonly discovered in DLBCL, like ATM (Ataxia telangiectasia mutated) mutations and deletions, MDM2 (murine double minute 2) deletions and ARF (alternate reading frame of CDKN2A locus) loss may induce p53 dysfunction despite unaltered gene^31^. To add more complexity to the issue, it has been shown in CLL, that patients harboring bi-allelic loss of p53 function have a dismal prognosis^32^. Recently a large comprehensive study revealing the molecular subtypes of DLBCL, verified the same phenomena in DLBCL as well^33^. Together these facts imply that a broader approach discovering the genetic landscape of the disease should be preferred in the future.

Patients with *TP53* mutations had a high frequency of primary refractory diseases. An interesting finding in our data was, however that among patients with mutated *TP53* (n = 10), in whom primary treatment was successful, no relapses were detected. This might imply that these patients would possibly benefit from more intensive primary treatments or from different treatment strategies such as new targeted therapies, e.g. kinase inhibitors idelalisib or ibrutinib. In chronic lymphocytic leukemia, an effect of these drugs has been shown to be independent of functional *TP53* genes^34,35^. In DLBCL ibrutinib has shown promise in treatment of ABC subtypes in a phase-2 trial^36^. These arising therapies warrant new studies to discover their therapeutic potential in high-risk DLBCL.

In our series *TP53* mutation frequency was lower than previously reported, i.e. 12.5% versus 20%, and this difference might be explained by selection bias, because small samples were excluded from the study^20^. Although difficulties were expected with sequencing of paraffin-embedded samples, the total success rate of sequencing was not optimal. To improve the success ratio, we excluded small biopsy samples, e.g. core needle samples, and only selected the exons that harbour most of the functional mutations. In addition, we divided exons 5 and 8 into two parts to improve the output.

Because gene sequencing is a challenging method to apply to routine clinical practice, it would be attractive to use IHC as a surrogate marker to find the mutated cases. We found that high p53 protein expression correlated with *TP53* gene mutations. However, it did not have statistically significant prognostic value, and half of the cases with strong expression had wild type p53 gene. These findings imply that p53 immunohistochemistry might be used for screening of the mutations but is not able to substitute sequencing^20^.

Here we report results of p53 gene sequencing, MYC, BCL-2 and BCL-6 FISH as well as MYC, BCL-2, BCL-6 and p53 immunohistochemistry in a moderate group of 155 DLBCL cases. Our data suggest that *TP53* mutations in LSH and L3, and IHC high expression of BCL-2 and MYC are each independently associated with poor prognosis in patients with DLBCL. The impact of p53 mutations was limited. Together with other existing data, this implies, that in the future studies also the existence of other wild type gene should be taken into account. Although we had a moderate patient population, considering the excellent prognosis of these patients, few events; relapses and disease related deaths occurred. Combining this fact with the rarity of studied molecular features we could not do detailed subgroup analysis and the results should be addressed with caution. However, most of the published studies face this same problem, which should therefore be addressed in a meta-analysis combining several studies. Despite all these limitations we found our study adds knowledge to this field of prognostic impact of molecular events in DLBCL.

## Methods

### Patients and samples

Paraffin-embedded tissue blocks from diagnostic lymph nodes or extralymphatic tumor-site samples were available from 155 untreated patients with histologically confirmed *de novo* DLBCL, not otherwise specified. Core needle biopsy samples were excluded on the basis of sample size. Detailed patient information was collected retrospectively in each case. Patients were diagnosed and treated at Oulu and Kuopio University Hospitals and Central Hospital of Central Finland between the years 2003–2011. The patient material was collected from three primary treatment facilities in Finland and overall treatment was uniform in all hospitals. Diagnoses were reviewed by experienced hematopathologists (KMH, YS and JB). Hans’ IHC algorithm was used to stratify DLBCL cases into GC and non-GC phenotypes^2^. The diagnostic work-up included medical history, physical examination, blood chemistry, bone marrow biopsy, and whole body computed tomography. Primary treatment for all patients was CHOP-like therapy combined with rituximab. The Ethics Committee of the Northern Ostrobothnia Hospital District approved the study design (Approval Number 42/2010, date 23 June 2010). The ethics committee waived the need to obtain informed consent. All experiments were performed in accordance with relevant guidelines and regulations.

### Microdissection and DNA isolation

DNA was obtained from paraffin-embedded tissue sections. Sections were cut into 10 µm-thick slices and mounted on polyethylene naphthalate (PEN) membrane-coated slides (P.A.L.M. Microlaser Technologies, Germany). Tissues sections were analyzed by experienced hematopathologists (KMH and JB) and areas with tumor tissue were marked and cut out using the P.A.L.M. Robot-microlaser system (P.A.L.M. Microlaser Technologies) with assistance of pressure catapulting according to the instructions of the manufacturer.

### *TP53* mutation analysis

Direct sequencing (ABI3130 Genetic Analyzer, Applied Biosystems, CA, USA) of tumor-derived DNA was performed for *TP53* exons 5, 6, 7 and 8 based on the sequence information (NG_017013.2, NM_000546.5) obtained from the NCBI public database. Six primer pairs (Table 4) were used in the PCR amplification with AmpliTaq-Gold® (Applied Biosystems) and in BigDye terminator v.1.1 cycle sequencing reactions (Applied Biosystems). For exons 5 and 8, two sets of primers were used in order to keep the PCR product sizes small (<200 bp) and thus suitable for sequence analysis of fragmented DNA. The PCR and sequencing reaction conditions are available upon request. All the sequencing reactions were carried out in both forward and reverse directions and any unclear results were confirmed by re-sequencing of the sample. PCR products were purified using ExoSAP-IT® (Affymetrix) or ExoStar™ (Illustra) one-step cleanup reactions. The sequencing reaction cleanup was performed with basic ethanol/EDTA precipitation. All the sequence data was analyzed with CodonCodeAligner v4.1.1 (CodoneCode Corporation) and Sequence Scanner v1.0 (Applied Biosystems) software. IARC database version R18, April 2016 was used to analyze the mutational data^37^.

**Table 4:** Primers for PCR and sequencing of *TP53* exons 5, 6, 7 and 8.

Primer	Sequence 5′ – 3′	PCR product size
Ex5A forward	CCTGACTTTCAACTCTGTCTC	158 bp
Ex5A reverse	ACTGCTTGTAGATGGCCATG	
Ex5B forward	CAGCTGTGGGTTGATTCCAC	182 bp
Ex5B reverse	CTGGGGACCCTGGGCAAC	
Ex6 forward	GCCTCTGATTCCTCACTGAT	181 bp
Ex6 reverse	TTAACCCCTCCTCCCAGAGA	
Ex7 forward	AGGCGCACTGGCCTCATCTT	177 bp
Ex7 reverse	TGTGCAGGGTGGCAAGTGGC	
Ex8A forward	CCTTACTGCCTCTTGCTTCTC	130 bp
Ex8A reverse	CTTGCGGAGATTCTCTTCCTC	
Ex8B forward	TTGTGCCTGTCCTGGGAGAG	127 bp
Ex8B reverse	CTCCACCGCTTCTTGTCCT	

### IHC staining and FISH

Immunostaining and fluorescence *in situ* hybridization (FISH) analyses were performed as previously described^25,38,39^. For these stainings, tissue microarrays were constructed^40^.

For IHC the following monoclonal antibodies were used in accordance with the manufacturer’s instructions. Monoclonal Rabbit Anti-Human c-MYC, clone EP121, dilution 1:100, Epitomics, CA, USA; Monoclonal Mouse Anti-Human BCL-2, clone124, dilution 1:100, Flex, Dako, Denmark; Monoclonal Mouse Anti-Human BCL-6, clone PG-B6p, RTU, Flex+, Dako, Denmark: Monoclonal Mouse Anti-Human p53 protein, clone DO-7, Flex, Dako, Denmark.

MYC, BCL-2 and BCL-6 protein expression was evaluated as a percentage of cells stained in 10-unit intervals. Previously described cut-off values were used for regards BCL-6 (50%), MYC (40%), BCL2 (70%)^29^ and p53 (50%)^39^.

Cut-off values were used to divide patients into high- and low-expression groups. Double-expressor (DE) lymphomas were defined as lymphomas with high expression irrespectively to the existence of gene translocations. The cut-off value used were BCL-6 staining over 50%, BCL-2 staining over 70% of the cells positive. For MYC IHC we performed analyses with both the cut-off value of 70% and 40%. The results are given mainly with the latter one.

The following FISH probes were used in accordance with the instructions of the manufacturer. *BCL2* FISH DNA Probe, Split Signal, Code Y5407, Dako, Denmark; *BCL6* Breakapart probe, LPH 035, Cytocell, United Kingdom; *MYC* FISH DNA Probe, Split Signal, Code Y5410, Dako, Denmark.

DH lymphomas were defined as those with concurrent *MYC* and *BCL-2* or *BCL-6* translocation. Triple-hit lymphomas were defined as lymphomas with *MYC* translocation combined with both *BCL-*2 and *BCL-6* translocation.

### Statistical analysis

Associations between the different variables and clinical parameters were assessed by using Pearson’s 2-sided chi-square test. Kaplan–Meier analyses were used to assess survival rates and log-rank tests were used to determine the statistical significance. Disease-specific survival (DSS) was calculated from the date of diagnosis to the date of lymphoma-related death or the last follow-up date. Overall survival (OS) was calculated from the date of diagnosis to death from any cause or last follow-up. Disease-free survival (DFS) was calculated from the date of diagnosis to the date of relapse or date of death from any cause, or last follow-up date, whichever occurred first. p-values < 0.05 were considered significant. To evaluate the independent prognostic potential, all significant associations with survival in univariate analysis were analyzed by means of Cox regression using the enter method. The model included International Prognosis Index (IPI) divided into three categories according to risk, lactate dehydrogenase, Eastern Cooperative Oncology Group (ECOG) performance status, Ann Arbor stage, age, B-symptoms and extranodal involvement. The three IPI categories were as follows: low 0–1, intermediate, 2–3 and high risk, 4–5. Lactate dehydrogenase categories were normal and high. ECOG performance status categories were 0 or 1 and 2, 3 or 4. Ann Arbor-stage categories were Stages I–II and III–IV. Age categories were under 60 and 60 or more. B-symptom categories were no and yes. Extranodal involvement was divided to no extranodal disease or extranodal involvement. All statistical analyses were performed using the Statistical Package for the Social Sciences, v. 22.0 (IBM SPSS, Chicago, IL, USA).

The datasets generated during and/or analysed during the current study are available from the corresponding author on reasonable request.
